# Predictive role of multiple gene alterations in response to cetuximab in metastatic colorectal cancer: A single center study

**DOI:** 10.1186/1479-5876-10-87

**Published:** 2012-05-08

**Authors:** Paola Ulivi, Laura Capelli, Martina Valgiusti, Wainer Zoli, Emanuela Scarpi, Elisa Chiadini, Paola Rosetti, Sara Bravaccini, Daniele Calistri, Luca Saragoni, Andrea Casadei Gardini, Angela Ragazzini, Giovanni Luca Frassineti, Dino Amadori, Alessandro Passardi

**Affiliations:** 1 Biosciences Laboratory, IRCCS Istituto Scientifico Romagnolo per lo Studio e la Cura dei Tumori (I.R.S.T.), Via Maroncelli 40, 47014, Meldola (FC), Italy; 2 Department of Medical Oncology, IRCCS Istituto Scientifico Romagnolo per lo Studio e la Cura dei Tumori (I.R.S.T.), Via Maroncelli 40,, 47014, Meldola (FC), Italy; 3 Unit of Biostatistics and Clinical Trials, IRCCS Istituto Scientifico Romagnolo per lo Studio e la Cura dei Tumori (I.R.S.T.), Via Maroncelli 40, 47014, Meldola (FC), Italy; 4 Pathology Unit, Morgagni-Pierantoni Hospital, Forlì, Italy

**Keywords:** Metastatic colorectal cancer, Cetuximab, KRAS, BRAF, PIK3CA, PTEN

## Abstract

**Background:**

*KRAS* mutations negatively affect outcome after treatment with cetuximab in metastatic colorectal cancer (mCRC) patients. As only 20% of *KRAS* wild type (WT) patients respond to cetuximab it is possible that other mutations, constitutively activating the EGFR pathway, are present in the non-responding *KRAS* WT patients. We retrospectively analyzed objective tumor response rate, (ORR) progression-free (PFS) and overall survival (OS) with respect to the mutational status of *KRAS*, *BRAF*, *PIK3CA* and PTEN expression in mCRC patients treated with a cetuximab-based regimen.

**Methods:**

67 mCRC patients were enrolled onto the study. DNA was extracted from paraffin-embedded sections derived from primary or metastatic lesions. Exon 2 of *KRAS* and exon 15 of *BRAF* were analyzed by direct sequencing, *PIK3CA* was evaluated by pyrosequencing and PTEN expression by immunohistochemistry.

**Results:**

*BRAF* and *PIK3CA* mutations were independently associated with worse PFS (*p* = 0.006 and *p* = 0.028, respectively) and OS (*p* = 0.008 and *p* = 0.029, respectively). No differences in clinical outcome were found between patients who were positive or negative for PTEN expression. Conversely, patients negative for *KRAS*, *BRAF* and *PIK3CA* mutations were characterized by significantly better ORR, PFS and OS than patients with at least one of these mutations.

**Conclusions:**

*BRAF* and *PIK3CA* mutations would seem to be independent predictors of anti-EGFR therapy effectiveness and could be taken into consideration during treatment decision making.

## Background

Colorectal cancer (CRC) is the third most common form of cancer and the second leading cause of cancer-related death. Although early diagnosis may allow radical surgery to be performed and result in a complete cure, about 25% of patients present with metastatic disease at diagnosis and about 40-50% of resected patients will develop distant metastases and die [1]. There is evidence that the use of polychemotherapy with fluoropyrimidines, oxaliplatin and irinotecan can significantly improve overall survival (OS) in patients with metastatic CRC (mCRC) with respect to those who do not receive all three drugs. The use of bevacizumab in association with chemotherapy has also been shown to prolong OS [2].

Current treatment options for mCRC include cetuximab (CTX), a chimeric IgG1 monoclonal antibody which binds to the epidermal growth factor receptor (EGFR), leading to inhibition of its downstream signaling. However, objective response rates (ORRs) in unselected mCRC populations are only around 8–12% for CTX when used in monotherapy [2-5]. As a number of retrospective studies have shown that somatic mutations of *KRAS* can negatively affect the efficacy of CTX [6-8], the use of the drug has been restricted by health authorities to patients with wild type (WT) *KRAS*. Despite this, relatively few patients benefit from CTX: ORRs are around 13% (*vs* about 1% in *KRAS* mutated) for monotherapy [9] and about 60% (vs 35% in *KRAS* mutated) when combined with chemotherapy [10,11]. These findings clearly suggest that other resistance mediators exist in non-responding WT patients. The predictive value of additional mutations and deregulations of signaling pathways downstream of EGFR such as *BRAFPIK3CA*, or PTEN is currently under intensive investigation.

BRAF plays a crucial role in the KRAS pathway and a key mutation (V600E) in exon 15 has been described in colon cancer [12,13]. A number of retrospective and preclinical studies have recently suggested that *BRAF* mutations are mutually exclusive with those of *KRAS* and may indicate resistance to anti-EGFR therapy in mCRC patients as well as in cellular models of CRC [14,15]. The *PIK3CA* gene is another downstream effector of *KRAS* and its pathway is normally inhibited by PTEN. The role of the PIK3CA/PTEN pathway in resistance to EGFR inhibitors has been investigated extensively in *KRAS* WT patients and cellular models of CRC, with conflicting results [16-22].

We retrospectively analyzed the relation between ORR, progression-free survival (PFS) and OS and the mutational status of *KRAS*, *BRAF*, *PIK3CA* and PTEN expression in mCRC patients treated with a CTX-based regimen, with the aim of clarifying the relative contribution of these molecular alterations to clinical outcome.

## Methods

### Patient population and treatment regimens

We retrospectively analyzed 67 evaluable patients with EGFR-positive mCRC, consecutively treated with a CTX-based regimen at Istituto Scientifico Romagnolo per lo Studio e la Cura dei Tumori in Meldola, Italy, from March 2004 to October 2010. Inclusion criteria were pathological diagnosis of stage IV colorectal adenocarcinoma, age > 18 years, Eastern Cooperative Oncology Group performance status < 3. Patients treated before June 2009 were selected for CTX on the basis of EGFR expression alone as *KRAS* mutational status evaluation had still not been made mandatory by the Italian Regulatory Authority. All patients treated after June 2009 had tumors negative for *KRAS* mutations.

Data on patient characteristics, treatment and outcome were collected. Treatment was continued until disease progression or toxicity occurred, as per standard criteria. Clinical response was assessed every 8 weeks with complete radiological examination (CT or MRI scan) and was evaluated *a posteriori* according to Response Evaluation Criteria in Solid Tumors (RECIST) guidelines. Objective tumor responses were classified into partial response (PR), stable disease (SD) or progressive disease (PD). Patients with SD or PD were defined as non-responders. The ORR was defined as the fraction of patients with complete or partial response confirmed at ≥ 4 weeks after the initial response. Toxicity was evaluated according to National Cancer Institute Common Terminology Criteria for Adverse Events v 3.0 guidelines for each patient receiving at least one dose of study treatment.

The study was approved by the local Ethical Committee in accordance with the ethical standards laid down in the 1964 Declaration of Helsinki. All patients gave their written informed consent.

### Molecular analyses

Formalin-fixed paraffin-embedded (FFPE) tumor blocks were reviewed for quality and tumor content. DNA was extracted from 5-μM FFPE sections of primary or metastatic lesions containing at least 50% of tumor cells. Exon 2 of *KRAS* and exon 15 of *BRAF* genes were amplified by PCR using the following primers: forward 5’-GGT GAG TTT GTA TTA AAA GGT ACT GG-3’ and reverse 5’ GGT CCT GCA CCA GTA ATA TGC-3’ for *KRAS*, and forward 5’ TCA TAA TGC TTG CTC TGA TAG GA-3’ and reverse 5’- GGC CAA AAA TTT AAT CAG TGG A-3’ for *BRAF*. PCR products were purified using MiniElute PCR purification kit (Qiagen, Hilden, Germany) and then submitted to sequencing using BigDye Terminator 3.1 Reaction Cycle Sequencing kit (Applied Biosystems, Foster City, CA). Sequence reactions were purified using DyeEx 2.0 Spin kit (Qiagen) and separated by capillary electrophoresis with laser-induced fluorescence detection (3100 Genetic Analyzer, Applied Biosystems).

*PIK3CA* status was analyzed by pyrosequencing using anti-EGFR MoAb response (*PIK3CA* status) (Diatech, Jesi, Ancona, Italy), according to the manufacturer’s instructions. Reactions were run on a PyroMark Q96 ID (Qiagen). PTEN protein expression was analyzed by immunohistochemistry using a Dako monoclonal antibody diluted 1:100. Samples with ≥ 5% immunopositive neoplastic cells of any intensity in cytoplasm and/or nucleus were considered as PTEN-positive.

### Statistical analyses

A two-sided Fisher's exact test was used to evaluate the association between mutations and ORR. PFS was calculated from the first day of treatment to the date of first observation of disease progression or last follow-up or death in the absence of progressive disease. OS was calculated from the first day of treatment to the date of death of any cause, or last follow-up. PFS, OS and their 95% confidence intervals (95% CI) were estimated using the Kaplan-Meier life-table method [23] and survival curves were compared by the logrank test [24].

Logistic regression was used to estimate the odds ratio of response to therapy and the 95% CI for mutational status in univariate analysis. Hazard ratios (HR) and their 95% CI were estimated according to Cox multiple regression model to evaluate the independent predictive role of mutational status of *KRASBRAFPIK3CA* and PTEN expression in PFS and OS [25]. Statistical significance was assumed for *p* < 0.05. Statistical analyses were carried out with SAS Statistical software (version 9.1, SAS Institute, Cary, NC).

## Results

### Molecular alterations

Among the 39 patients treated with CTX-based treatment before June 2009 we detected 14 (36%) *KRAS* mutations, whereas all 28 patients treated after June 2009 had WT *KRAS.* In the overall series, 10 (71%) *KRAS* mutations occurred in codon 12, of which 4 were G12V, 3 G12S, 2 G12D and 1 G12A alterations. In 4 (29%) cases mutations occurred in codon 13 and were all G13D alterations. *BRAF* mutations were detected in 12 (17.9%) patients and all were V600E alterations. Mutations in the *PIK3CA* gene were detected in 9 (13.4%) patients involving exon 9 (4 E545K, 2 E542K, 1 E545G) in 7 cases and exon 20 (both H1047R) in 2 cases. Loss of PTEN expression was observed in 40 (59.7%) cases.

**Figure 1:**
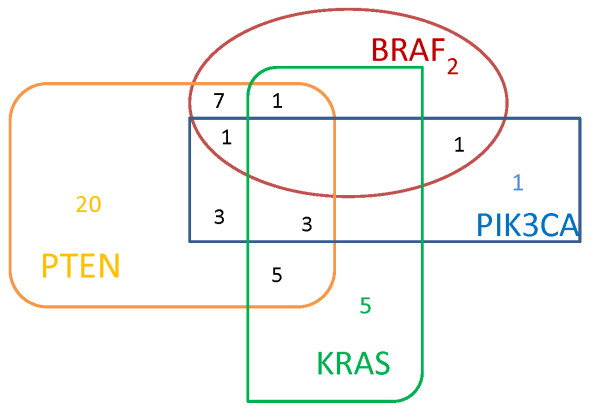
**Distribution of different molecular alterations in individual tumors of the 67 patients. ***KRAS* and *BRAF* mutations occurred in a mutually exclusive manner in all but one patient, while an overlapping pattern was observed among the other gene alterations.

*KRAS* and *BRAF* mutations occurred in a mutually exclusive manner in all but one patient, while an overlapping pattern was observed among the other gene alterations. We observed only one mutation in 28 cases, two overlapping mutations in different combinations in 16 cases and three overlapping alterations in 5 cases. The most frequent overlapping alterations were *BRAF*/PTEN (9 cases), *PIK3CA*/PTEN (7 cases) and *KRAS*/*PIK3CA*/PTEN (3 cases) (Figure 1).

### Clinical variables

Clinical characteristics of patients are shown in Table 1. Our cohort included heavily pretreated patients, more than 75% of whom had received at least 2 lines of chemotherapy for metastatic disease, including irinotecan, oxaliplatin and fluoropyrimidines, and in 38.8% of cases, bevacizumab. The vast majority of patients (89.5%) were treated with irinotecan-based chemotherapy plus CTX. Overall there were 17 responders (ORR 25.4%) and 50 non responders (74.6% of whom 28.3% SD and 46.3% PD). Analysis of clinical variables showed that only cutaneous toxicity (2–3 *vs* 0) and ECOG PS (1–2 *vs* 0) were associated with significantly better median PFS (*p* = 0.014 and 0.0007, respectively). Other clinical variables including gender, site of primary tumor (colon, rectum), age and number of previous cancer treatments for advanced disease were not predictors of clinical outcome.

**Table 1:** Baseline patient characteristics

	***n*** **(%)**
**No. of patients**	67
**Median age, yrs** (range)	61 (34–79)
**Gender** (male/female)	
Male	39 (58.2%)
Female	28 (41.8%)
**Performance Status**	
0	37 (55.2%)
1-2	30 (44.8%)
**Primary tumor site**	
Colon	54 (80.6%)
Rectum	13 (19.4%)
**Treatment regimen**	
CTX + irinotecan/folfiri	60 (89.5%)
CTX + FOLFOX4	6 (9.0%)
CTX alone	1 (1.5%)
**Previous chemotherapy**	
Irinotecan-based	62 (92.5%)
Fluoropyrimidine-based	67 (100%)
Oxaliplatin-based	54 (80.6%)
Bevacizumab-based	26 (38.8%)
**No. of previous cancer treatments for advanced disease**	
One	15 (22.4%)
Two	28 (41.8%)
Three	15 (22.4%)
More than three	9 (13.4%)
**Cutaneous toxicity**	
0	19 (32.8%)
1	19 (32.8%)
2–3	20 (34.5%)
Unknown	9

### Molecular alterations and clinical outcomes: Univariate analyses

The role of each molecular alteration is shown in Table 2. We found a better, albeit not statistically significant, ORR in patients with WT tumors with respect to those with *KRAS*, *BRAF*, or *PIK3CA* mutations, while no differences were found in PTEN-positive compared to PTEN-negative patients. At a median follow-up of 28 months, 61 cases of progressive disease and 57 deaths had been registered. Median PFS and OS were significantly better in patients with wild type *BRAF* and *PIK3CA*. Only a trend towards a better median PFS was observed in patients with wild type *KRAS* or with high PTEN expression.

**Table 2:** Biomolecular alterations and ORR, PFS and OS: univariate analysis

	***n***	**ORR %**	**Median PFS (months)** **(95% CI)**	***p***	**Median OS (months)** **(95% CI)**	***p***
**Overall**	67		4.3 (2.9-5.5)	-	9.2 (7.3-12.0)	-
***KRAS***						
WT	53	30.2	5.2 (3.4-6.7)		8.7 (6.9-14.6)	
Mut	14	7.1	2.7 (2.2-3.9)	0.070	9.4 (6.0-12.0)	0.114
***BRAF***						
WT	55	29.1	5.1 (3.2-6.7)		9.6 (8.3-13.9)	
Mut	12	8.3	2.8 (1.4-3.9)	0.005	5.8 (2.1-8.4)	0.008
***PIK3CA***						
WT	58	27.6	5.1 (3.4-6.2)		9.9 (8.3-13.7)	
Mut	9	11.1	2.3 (2.1-3.3)	0.031	6.6 (4.4-7.3)	0.013
**PTEN**						
<5%	40	22.5	3.3 (2.3-5.2)		8.3 (6.0-12.4)	
≥5%	27	29.6	6.2 (4.0-8.7)	0.073	11.0 (8.0-14.6)	0.647

### Molecular alterations and clinical outcomes: Multivariate analyses

Multivariate analysis was performed on all 4 molecular alterations and was adjusted for PS, cutaneous toxicity, and number of previous chemotherapy lines. No correlation was found between mutation status and lack of objective response. The analysis did, however, confirm, that wild type *BRAF* and *PIK3CA* were significantly and independently associated with better PFS [HR = 2.65 (95% CI 1.33-5.29), *p* = 0.006 and HR = 2.46 (95% CI 1.10-5.52), *p* = 0.028, respectively], with *KRAS* mutations also exerting a detrimental borderline effect [HR = 1.86 (95% CI 0.99-3.47), *p* = 0.052]. Conversely, PTEN expression was not correlated with PFS. With regard to OS, *BRAF* and *PIK3CA* mutations were once again associated with decreased survival [HR = 2.47 (95% CI 1.26-4.85), *p* = 0.008 and HR = 2.51 (95% CI 1.10-5.72), *p* = 0.029, respectively], whereas *KRAS* and PTEN alterations did not independently affect clinical outcome (Table 3).

**Table 3:** Biomolecular alterations and ORR, PFS and OS: multivariate analysis

	**PFS** **HR (95% CI)**	***p***	**OS** ** HR (95% CI)**	***p***
*KRAS* (mutated *vs* wild type)	1.86 (0.99-3.47)	0.052	1.56 (0.83-2.96)	0.170
*BRAF* (mutated *vs* wild type)	2.65 (1.33-5.29)	0.006	2.47 (1.26-4.85)	0.008
*PIK3CA* (mutated *vs* wild type)	2.46 (1.10-5.52)	0.028	2.51 (1.10-5.72)	0.029
PTEN (<5% *vs* ≥5%)	1.47 (0.85-2.54)	0.169	0.89 (0.50-1.57)	0.686

In accordance with the new patient selection criteria for CTX, we analyzed the effect of *BRAF* and *PIK3CA* mutations and loss of PTEN on the 53 patients with wild-type *KRAS* tumors. At multivariate analysis, *BRAF* mutation was confirmed as a predictor of worse PFS [HR = 3.15 (95% CI 1.51-6.59), *p* = 0.002] and OS [HR = 2.87 (95% CI 1.37-6.00), *p* = 0.005] and *PIK3CA* mutations were correlated with a shorter, albeit not statistically significant, PFS, whereas no correlation was observed with respect to OS. Finally, PTEN expression was not found to affect either PFS or OS.

### Number of tumor molecular alterations and clinical outcome

The contribution of the number of mutations in determining the clinical outcome of patients was examined. In particular, we considered the combination of *KRAS*, *BRAF* and *PIK3CA* mutations, excluding PTEN expression because of its low impact as an independent predictive marker. Our results showed that 38 (56.7%) patients did not have any alterations, 23 (34.3%) had one mutation and 6 (9.0%) had 2 mutations.

**Figure 2:**
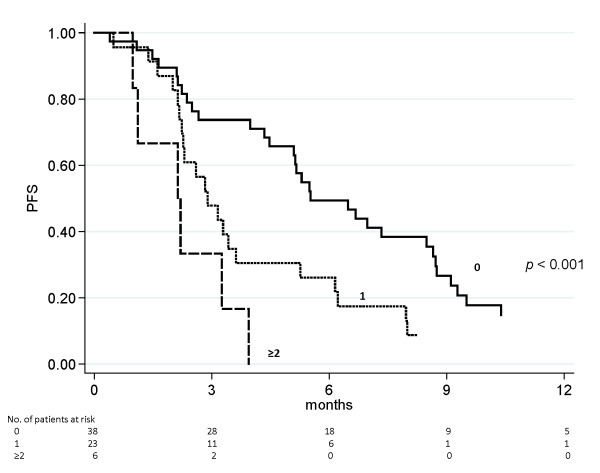
**PFS on the basis of the number of tumor molecular alterations.** A significant difference (*p* < 0.001) was observed among patients harboring no (5.5 months), one (2.9 months) or two (2.2 months) alterations.

**Figure 3:**
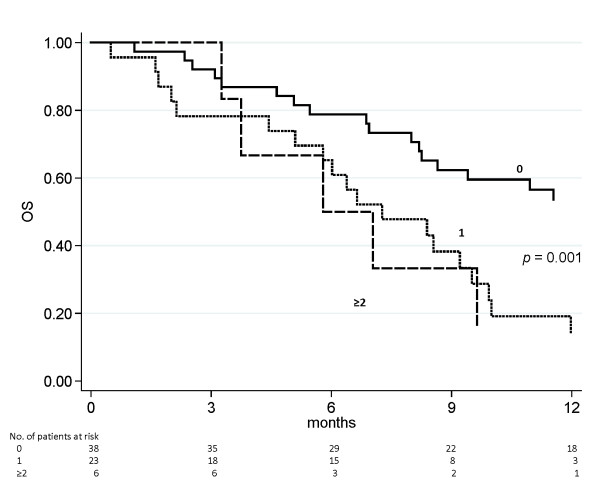
**OS on the basis of the number of tumor molecular alterations.** A significant difference (*p* < 0.001) was observed among patients harboring no (13.9 months), one (7.3 months) or two (6.4 months) alterations.

Objective response rates were 36.8% in ‘triple wild type’ patients and 13.0% in those with one mutation, while no responses were observed in patients with 2 mutations. The odds ratio of response was 0.20 (95% CI 0.05-0.77) (*p* = 0.020) in patients with at least one mutation compared to those with no mutations. Similarly, survival analysis showed that patients with at least one mutation had worse PFS and OS with respect to those with none. In particular, median PFS (95% CI) was 5.5 (5.1-8.7), 2.9 (2.3-3.6) and 2.2 months (1.1-3.3) for patients harboring no alterations, one or two alterations, respectively, *p* < 0.001 (Figure 2). Median OS (95% CI) was 13.9 (8.3-17.6), 7.3 (5.8-9.5) and 6.4 months (3.7-9.6) for patients harboring no alterations, one or two alterations, respectively, *p* = 0.001 (Figure 3).

## Discussion

The clinical impact of monoclonal antibodies targeting EGFR in patients with mCRC has been clearly established. In particular CTX, alone or in combination with conventional chemotherapy, has been shown to improve the outcome of patients treated in first-, second- and third-line settings. *KRAS* mutational status is currently a validated predictive biomarker used to select mCRC patients for EGFR-targeted drugs, such as CTX and panitumumab. However, response rates to either drug are less than 20% in wild-type *KRAS* patients. Although recent reports have indicated that *BRAFPIK3CA* or PTEN alterations may constitute additional mechanisms of resistance to these drugs, results are still conflicting [14,15,19-22,26,27]. Our case series included patients with *KRAS* wt and *KRAS* mutated tumors treated with CTX before June 2009, and others selected with *KRAS* wt tumors treated after June 2009. We observed a better, albeit not statistically significant, ORR, OS and PFS in patients with *KRAS* wt tumors with respect to those with *KRAS* mutated tumors. This lack of statistical significance may be a result of the limited number of mutated cases due to the selection of wt patients after June 2009. It is also possible that a complex relationship exists between anti-EGFR response and KRAS status due to the synergistic effects of mutant and wild type KRAS proteins [28].

Data were recently published on a subgroup analysis of patients treated with chemotherapy and CTX (trial NCIC CTG CO.17), showing that those with tumors harboring *KRAS* G13D mutations (14.5% of the *KRAS* mutated group) had better PFS and OS compared to patients with other *KRAS* mutations. No significant differences in PFS and OS were noted when patients with *KRAS* G13D mutations were compared with those with wild type *KRAS* tumors [29]. In our experience 4 patients with *KRAS* G13D mutation did not respond. No correlations can be made with other types of mutations because of the low number of such mutations found. However, of the four patients with the G12V mutation, which is considered the most aggressive *KRAS* alteration in colorectal cancer [30,31], only one showed a partial response.

Significantly worse OS and PFS were observed in *BRAF* or *PIK3CA* mutated patients, suggesting that these two alterations may play an important role in determining anti-EGFR resistance. In particular, we found a high incidence of *BRAF* mutations (17.9%), possibly due to the high prevalence of wild type *KRAS* tumors which more frequently harbor alterations of the *BRAF* gene. It has been demonstrated that an activating mutation in *BRAF* is associated with resistance to treatment with anti-EGFR antibodies [14,15]. However, other studies have reported that *BRAF* mutations appear to be prognostic rather than predictive as mCRC patients not receiving CTX also have markedly reduced survival when tumors harbor a *BRAF* mutation [32-34]. All these studies suggest that a *BRAF* mutation precludes benefit from any type of treatment. Similarly, our multivariate analysis on *KRAS* wt patients confirmed *BRAF* mutation as a significant predictor of worse PFS and OS following CTX-containing treatment.

Conflicting results have been obtained for *PIK3CA* mutations and CTX response [6,21,33,35,36]. In our study exon 9 and 20 mutations of the gene were associated with significantly worse PFS and OS, suggesting that the constitutive kinase activity of the mutated protein may overcome the inhibition signal from CTX. Moreover, in the subgroup of *KRAS* wild type patients, *PIK3CA* status was significantly associated with a better PFS and with a better, albeit not statistically significant, OS.

We did not obtain significant results on PTEN alterations, in accordance with some studies [21,27] but in contrast to others in which a correlation was observed between the lack of PTEN expression and response to CTX [19,37]. The discordance between the different immunohistochemical studies could be due to a number of factors, *e.g.* a lack of standardization of the reagents and protocols used; the absence of a single cut off value; or the subjective interpretation of the operators who evaluated the samples from a morphological point of view. As the best cut off to define PTEN positivity has not been clearly established, we used a value of 5%.

## Conclusions

Our study shows that *BRAF* and *PIK3CA* mutations are independently associated with worse ORR, PFS and OS, and that patients with wild type *KRAS*, *BRAF* and *PIK3CA* status have a significantly better outcome with respect to patients with at least one alteration. These results suggest that *BRAF* and *PIK3CA* could also be taken into consideration in the decision making for CTX-based treatment. Having said that, the number of patients in the present work is too small to reach any definitive conclusions, and larger prospective studies are now needed to validate our findings. Moreover, it would be opportune to confirm the results in a series of patients not treated with CTX in order to exclude the possibility that *BRAF* and *PIK3CA* mutations have a prognostic rather than predictive value.

## Abbreviations

CRC: Colorectal cancer; mCRC: Metastatic colorectal cancer; CTX: Cetuximab; ORR: Overall response rate.

## Competing interests

The authors declare that they have no competing interests.

## Authors' contributions

PU and AP conceived the study, participated in its design and drafted the manuscript. LC, EC, SB carried out the molecular analyses. MV, PR, ACG, AR made substantial contributions to acquisition of data. WZ, DC, GLF, DA made substantial contributions to the analysis and interpretation of data. ES performed the statistical analyses. LS revised all histological samples.

All authors read and approved the final manuscript.
